# Detection of Motion on a Trampoline with a Smartwatch

**DOI:** 10.3390/s21248413

**Published:** 2021-12-16

**Authors:** Satoshi Kobayashi, Tatsuhito Hasegawa

**Affiliations:** Graduate School of Engineering, University of Fukui, Fukui 910-8507, Japan; t-hase@u-fukui.ac.jp

**Keywords:** human activity recognition, trampoline exercise, smartwatch

## Abstract

In this study, we develop a method for detecting the motions performed on a trampoline using an accelerometer mounted on a smartwatch. This method will lead to a system that can be used to promote trampoline exercise using a home trampoline by detecting motions on the trampoline using a smartwatch. We proposed a method based on the convolutional neural network to detect the motions on a trampoline. As a result of the performance evaluation by leave-one-subject-out cross-validation on eight subjects, our method achieves 78.8% estimation accuracy, which is the best estimation accuracy compared to the baseline methods. We also evaluate the inference time and the battery consumption when the model is actually running on a smartwatch. Our method is effective for on-device prediction.

## 1. Introduction

The demand for home exercise is increasing, as evidenced by the strong sales of Ring Fit Adventure [1], which combines games and exercise to provide continuous exercise. In addition, moderate exercise in daily life is said to be necessary for the prevention of lifestyle diseases [2]. Therefore, it is important to have a means to continuously exercise in a manner appropriate for one’s physical fitness.

Aerobics performed on a trampoline is called “trampobics”, and it is attracting attention as a form of training [3]. Aerobics on a hard floor may cause damage to the legs and back due to strenuous movements, whereas that on a trampoline can reduce the burden on the legs and back. In addition, trampoline exercise is perceived to be easier in subject evaluation than its equivalent exercise on flat ground, albeit the amount of exercise being the same [4]. However, exercise for the mere purpose of health maintenance tends to lose the motivation to continue and does not become a habit [5]. Trampoline exercise is considered to be monotonous and lacks continuity. Some systems can be used to make trampoline exercise entertaining [6,7]. Some studies have developed systems to promote trampoline exercise [8,9,10]. However, these systems are not easy to use because they require visiting a facility or using a dedicated device, which takes cost for dedicated equipment.

In recent years, various sensors have become readily available due to the widespread use of smartphones and smartwatches. Many studies have used the sensor data obtained from smart devices, such as smartphones and smartwatches. In particular, there have been studies on estimating human activity from sensor data [11,12,13,14,15].

**Figure 1:**
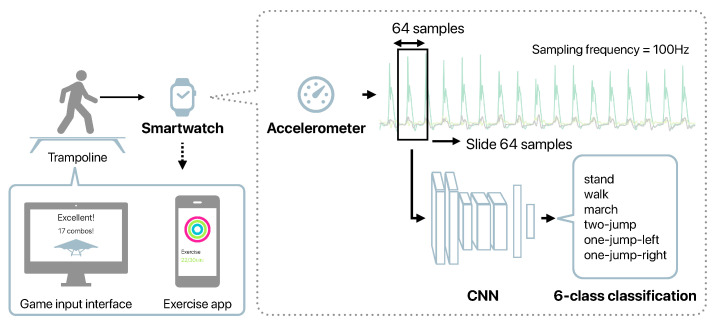
Outline of the proposed system.

We aim to develop herein a system to promote trampoline exercise using a smartwatch and a home trampoline. This study verifies and reports whether it is possible to detect the motions performed on the trampoline by a smartwatch, and whether it is possible to run on a real smartwatch. Figure 1 shows the outline of the proposed system. The motion sensor data obtained from the smartwatch worn on the arm were used to estimate a motion on the trampoline. Compared to smartphones, smartwatches can be worn at a fixed position and can easily be fixed; hence, they do not interfere with the trampoline exercise. The estimation results obtained from a smartwatch can be used as the game interface, and the exercise can be recorded with detailed information. Controlling a game by the motions performed on a trampoline can increase the motivation for trampoline exercise and improve sustainability. We developed a method for estimating the motions performed on a trampoline from a smartwatch accelerometer, which is the core of the proposed system, and show its effectiveness through experiments.

## 2. Related Works

### 2.1. Systems for Promoting Trampoline Exercise

Fukuchi et al. [8] developed a system to promote trampoline exercise by adding a selfie camera function. In this system, a distance sensor is placed under the trampoline to automatically take a picture of the user jumping on the trampoline. The time to reach the top is then estimated from the jumping interval. Meanwhile, Mori et al. [9] developed a system that changes the image on the virtual reality by the motions on the trampoline. This system detects the walking, balancing, and jumping motions on the trampoline using the two-dimensional information of the trampoline surface, which is measured by a non-contact infrared distance sensor. Mukai et al. [10] developed a motion detection method to be used as an interface for trampoline games. They proposed a detection method using a laser range scanner and a distance sensor, and verified its effectiveness in a treasure-hunting game.

HOP AMP [6] is a system that makes a trampoline entertaining. It measures the user’s jumping state from a mechanical sensor under the trampoline surface and changes the image projected on the trampoline surface by the user’s jumping state. Space Hoppers [7] is a trampoline attraction. In this game, players win a race by jumping on the trampoline. Sensors at the bottom of the trampoline measure the landing position. The player can accelerate in the game when he/she jumps high in the center of the trampoline.

### 2.2. Motion Sensor-Based Human Activity Recognition

Many studies focused on recognizing human activity from the motion sensor (e.g., accelerometers). Machine learning is often used for human activity recognition, and many studies have applied deep learning [12,13,14]. Hasegawa et al. [15] evaluated whether or not the model structure of the convolutional neural network (CNN), which is often used in image recognition field, is also effective in human activity recognition. In addition, due to the availability of public datasets, such as the OPPORTUNITY [16,17] and the HASC dataset [18], many studies have discussed human activity recognition using sensor data.

### 2.3. On-Device Deep Learning

In general, increasing the number of layers in a deep learning model increases the number of parameters and improves the model representation. However, increasing the number of layers increases the computational cost and requires a large amount of computational resources. In the image classification field, the model structures that achieve high accuracy while reducing the number of parameters, such as MobileNet [19] and EfficientNet [20], have been proposed. MobileNet was particularly developed as a model that can operate in environments with limited computing resources, such as smartphones.

Deep learning can be provided as an application in two ways: on a server (cloud) and on a device, such as a smartphone (on-device). In the case of cloud computing, a model is usually implemented on a server because it can use the large amount of computing resources on the server for prediction. In the case of on-device systems, the user’s privacy is protected; there is no need to connect to the Internet; and the server cost is not required [21]. However, smartphones have limited computational resources compared to servers; therefore, a model with a small computational cost is needed.

The frameworks for implementing deep learning models on smartphones, such as TensorFlow Lite [22], PyTorch Mobile [23], and Core ML [24], have been released since 2017. These frameworks enable the development of applications that use deep learning models without a server for prediction.

Deep learning models that work on smartphones or edge devices have been developed in the field of human activity recognition. Teng et al. [25] proposed a method for training a CNN model with small filters using a separate loss function for each layer. They also implemented the proposed model on an Android smartphone using PyTorch Mobile to evaluate the on-device prediction time. Xu et al. [26] proposed InnoHAR, a combination of the Inception module [27] and a gated recurrent unit (GRU). Moreover, they implemented the model in the MinnowBoard Turbot (MinnorBoard Wiki. MinnorBoard Turbot. http://minnowboard.outof.biz/MinnowBoard_Turbot.html (accessed on 27 April 2021)), which is a small embedded platform, and evaluated the prediction time. In addition, Agarwal et al. [28] proposed a lightweight deep learning model for human activity recognition that can run on an edge device.

### 2.4. Contributions of the Study

From the above information, the following are the main contributions of this study.

- We propose a method for detecting the motions on a trampoline using a smartwatch.
- We investigate how detailed the motion detection on the trampoline can be estimated from the acceleration data obtained from the smartwatch.
- We implement our method as an application that runs on a smartwatch, and evaluate its effectiveness in terms of the prediction time and the battery consumption.

We chose the motions that can be performed on a home trampoline, referring to papers that have examined the effects of trampoline exercises. We decided the following six types of motions: two-legged standing (ST; stand) [29], walking (WL, walk) [30], marching (jumping-walking) (MR; march) [29], two-legged jumping (TJ; two-jump) [30,31,32], one-legged jumping (left) (LJ; one-jump-left), and one-legged jumping (right) (RJ; one-jump-right) [31,33,34].

## 3. Method of Detecting Motions on a Trampoline with a Smartwatch

Figure 1 depicts the outline of our method. This system input is the 3-axis acceleration data obtained from a smartwatch worn on the arm. We proposed herein a CNN-based motion detection method. The measured acceleration data were divided into fixed windows to use as the CNN input. We divided the acceleration data into windows with a window size of 64 and a stride width of 64. When the sampling frequency was 100 Hz, the model predicted the motion performed on the trampoline from the data of 0.64 s.

**Figure 2:**
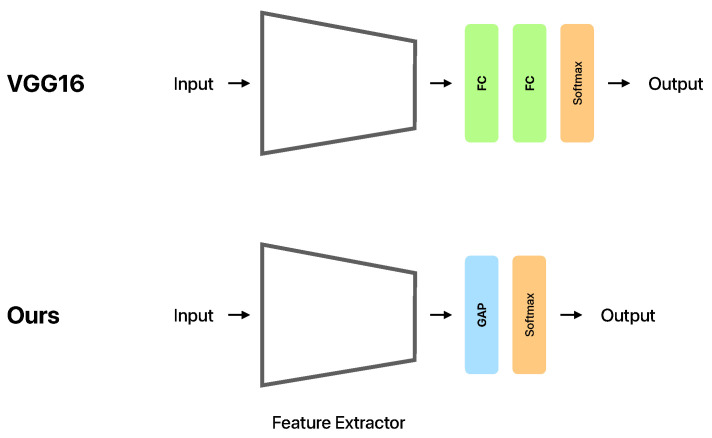
Overview of the original VGG16 and the model used in this study (Ours). The Feature Extractor denotes the part of the architecture shown in Table 1, from the first Conv1D with the 16 filters to the MaxPooling1D before the GAP. FC indicates the fully-connected layer. GAP indicates the global average pooling layer. Softmax is the output layer with softmax activation function.

Table 1 shows the CNN model used in this study. Conv1D indicates the convolutional layer. MaxPooling1D indicates the pooling layer. GAP indicates the global average pooling layer. The activation function of the output layer is the softmax function. Hasegawa et al. [15] showed that VGG16 [35] is a model that achieves a high estimation accuracy in human activity recognition; hence, we adjusted the parameters based on VGG16. As shown in Figure 2, global average pooling (GAP) [36] was used instead of fully-connected layers to reduce the number of parameters. We adopted GAP because reducing the number of parameters not only reduces the model size, but also suppresses overfitting. In addition, by reducing the number of parameters, it may have the effect of easily training the model in human activity recognition with limited training data.

## 4. Experiments for Evaluating the Classification Performance

### 4.1. Data Collection

We conducted a data collection experiment to evaluate the estimation accuracy of our method. A smartwatch was worn on the left wrist, and the user performed motions on a trampoline. One set consisted of the subjects moving in the order of ST, WL, MR, TJ, LJ, RJ for 10 s. Five sets were performed by each subject. The subjects took a 1 min rest between each set. The subjects were eight healthy males in their 20 s (Table 2). The height and weight information were self-reported and not actual measurements.

**Figure 3:**
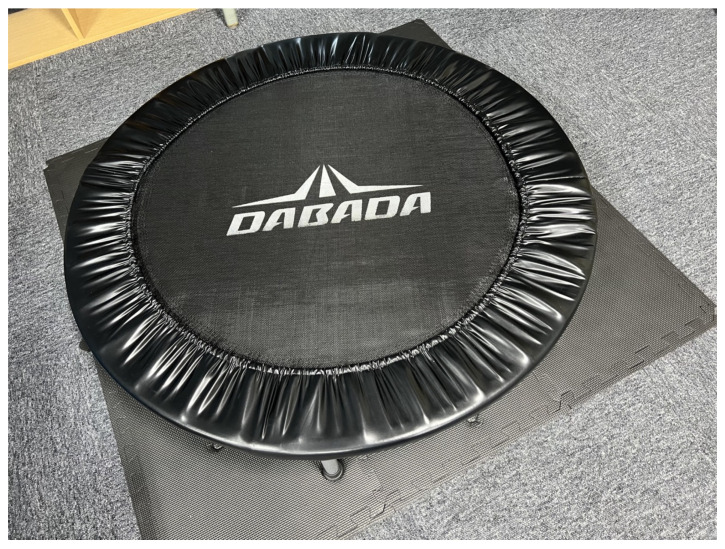
Home trampoline used in the experiments.

**Figure 4:**
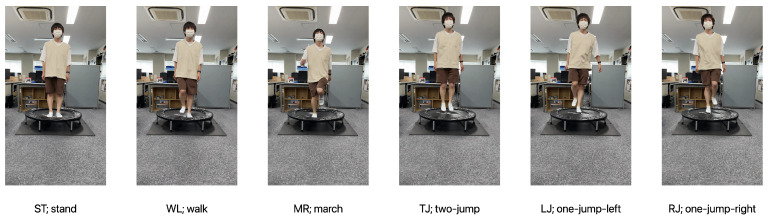
Six types of motions on the trampoline to be recognized by our method. ST is the staying behavior where the user stands and stops on the trampoline. WK is the walking behavior where the user stomps his/her feet on the trampoline. MR is also the walking behavior where the user stomps his/her feet while jumping lightly on the trampoline. TJ is the jumping behavior where the user jumps on the trampoline with both feet. LJ is also the jumping behavior where the user raises his/her right leg and jumps on the trampoline with his/her left leg. RJ is the opposite of LJ, the jumping behavior where the user raises his/her left leg and jumps on the trampoline with his/her right leg.

Figure 3 shows the home trampoline used in the experiments. In this experiment, we used a trampoline with a diameter of 102 cm (the diameter of the membrane surface is 67.5 cm). Figure 4 shows the six types of motions performed on the home trampoline show in Figure 3.

The smartwatch used for the data collection was an Apple Watch SE. We measured the data using a developed application that collects acceleration data. This application used the Core Motion framework [37], where the acceleration data unit is G. Therefore, −1.0 is observed in the Z axis when the screen was placed on a desk with the top up. In other words, $1\;\left[G\right]=9.8\;\left[{m/s}^{2}\right]$. The sampling frequency was set to 100 Hz. In the data collection experiment, we also collected the data from the Apple Watch gyroscope and the accelerometer, gyroscope, and magnetic sensor of the iPhone stored in the right front pocket of the pants. However, these data were not used in this study.

### 4.2. Baseline

As a baseline for the conventional method, we compared our method with the method using hand-crafted features (HCF) (Table 3) and Random Forest (RF) [38]. The features in Table 3 are extracted from the three-axis acceleration data and used as input to the RF. We adopted the features used in the study to estimate the smartphone position from its acceleration sensor [39] and the study to estimate the type of sidewalk surface [40]. For the frequency domain, we subjected the frame to a fast Fourier transform and calculated the same values in the all-, low-, mid-, and high-frequency regions. The low-frequency region was 0–4.2 Hz, the mid-frequency region was 4.2–8.4 Hz, and the high-frequency region was 8.4–12.6 Hz.

We also performed a comparison with the baseline CNN models: the simple CNN model proposed in the related work [14] (Simple CNN) and the original VGG16 using fully-connected layers [15] (VGG16).

### 4.3. Evaluation Method

We adopted accuracy as an evaluation index because the number of data for each class was not significantly biased. We used the leave-one-subject-out cross-validation (LOSO-CV) as the evaluation method. LOSO-CV used one subject as the test data and the remaining subjects as the training data, and replaces the test subject to test all the subjects.

The CNN models were optimized with Adam [41]. The learning rate was set to $1.0\times 10^{-3}$. The loss function was categorical cross entropy. The minibatch size was 20. The number of epochs was 100. We used TensorFlow to build the models. The models were trained on MacBook Pro (13-inch, M1, 2020) with 16 GB RAM.

## 5. Results

### 5.1. Estimation Accuracy

We compared the estimation accuracies of each method for each subject. Table 4 shows the accuracies for each subject. The accuracies in bold indicate the highest accuracy achieved for each subject. “Avg.” at the bottom of the table denotes the average estimated accuracy of all subjects. Table 4 presents that our method achieves the highest estimation accuracy on average for all subjects. Our method was more accurate than VGG16 when the fully-connected layers were replaced with GAP. However, some subjects achieved the highest accuracy with other methods. Subject D achieved the highest accuracy with the method using HCF and RF, suggesting that the CNN model was ineffective. In short, a difference existed in the effective features among individuals. However, our method works well for most of the subjects. Our method achieved the highest accuracy on average, and more than half of the subjects yielded the highest accuracy. In summary, our method was accurately estimated in most cases of detecting the motions performed on a trampoline.

### 5.2. Verification of Estimation Accuracy When the Number of Convolutional Layers Is Changed

Table 4 shows that Simple CNN with three convolutional layers can achieve almost the same accuracy as Ours. In human activity recognition, some studies have achieved adequate performance with a small CNN model [13,14]. Therefore, we also examine the effect of the number of convolutional layers on the accuracy in our method. The number of convolutional layers was increased from 1 to 19, with a pooling layer between every three layers. Also, the number of output channels in the convolutional layer started at 16, and was doubled every three layers.

**Figure 5:**
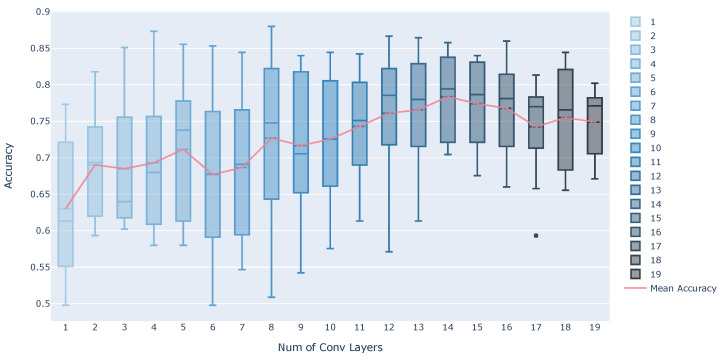
Estimation accuracy when the number of convolutional layers is changed.

Figure 5 shows the estimation accuracy when the number of convolutional layers is changed. The box plots are estimation accuracy for each subject, and the line chart is the mean of accuracy. According to Figure 5, the average accuracy tends to improve as the number of convolutional layers is increased. However, there is a slight tendency for the accuracy to decrease when the number of layers exceeds 15. The number of convolutional layers in Ours is 13, which mostly achieves better accuracy. If we see the results with three convolutional layers, which is the same as Simple CNN, we can find a discrepancy from the results in Table 4. This is probably due to the difference in the number of output channels in the convolutional layer and the number of pooling layers.

### 5.3. Possibility of Detailed Classification

Table 5 and Table 6 present the confusion matrix for RF and our method, respectively. Recall is the rate of the correctly predicted data in each class. Precision is the rate of the correctly predicted data out of all the predicted data. The F-measure is the harmonic average of recall and precision. The F-measure showed that both methods can estimate ST and WL with high accuracy. In addition, when our method was used, the number of cases of misclassifying three jump types was reduced when compared with that of the RF. The number of cases, in which LJ and RJ were misclassified, was reduced, suggesting that our method was effective in classifying jumps. However, both methods misclassified MR, LJ, and RJ in many cases, implying that it was difficult to classify these targets. This was because marching is a combination of left and right one-legged jumps; thus, it was difficult to distinguish them with the window width used herein (64 samples; 0.64 s).

### 5.4. Verification of the Effectiveness of Our Method in Practical Use

We discuss herein whether our method is effective in practical use. When training only with the data of other subjects, the estimation accuracy obtained by our method was 78.8% on average. The estimation accuracy was only approximately 70% for some subjects; hence, the usability of our method might decrease in practical use. In the case of activity recognition, the estimation accuracy is improved when the user’s own data are included in the training data. Core ML and TensorFlow Lite have functions for on-device personalization [42,43]. We now examine the estimation accuracy assuming a situation where three sets of user data are available for training.

**Figure 6:**
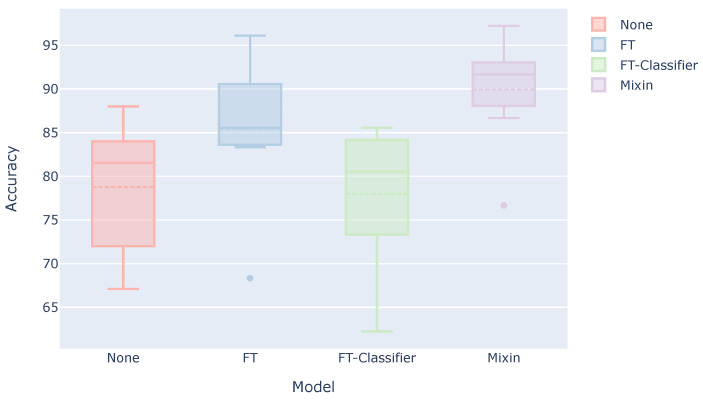
Estimation accuracy when using the user data for training.

Figure 6 shows a box plot of the estimation accuracy for each subject when three sets of test user data are used for the training. Each model is described as follows.

- None: Model trained using only the data of the other subjects (This result is the same as the “Ours” in Table 4).
- FT: Model trained using the data of other subjects and then additionally trained using the user’s three sets.
- FT-Classifier: Model trained using the data of other subjects and then additionally trained with only the classifier part using the user’s three sets.
- Mixin: Model trained using the combined data of other subjects and the user’s three sets.

FT-Classifier, in which only the classifier part was additionally trained, achieved the same estimation accuracy as None. On the contrary, the estimation accuracy is improved when the convolutional layers were also additionally trained. In general, deep transfer learning, which freezes the feature extractor, works well. However, in this case of personalization, the additional training of only the classifier did not necessarily work well, and it was also necessary to additionally train the feature extractor. In particular, since the classifier is composed of a single output layer, the number of parameters for personalization may have been too small. The accuracy will also be improved if the model is retrained by adding the user data to the training data.

**Figure 7:**
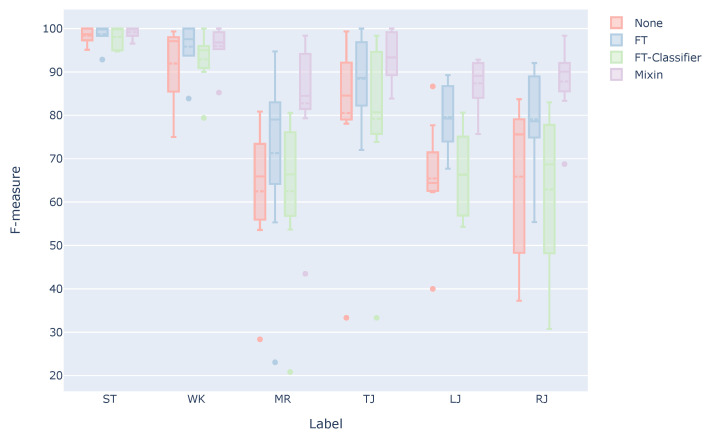
F-measure for each estimation target when using the user data for training.

Figure 7 shows a box plot of the F-measure. In the case of None, which is a model trained only with the data of other people, the F-measure of MR, LJ, and RJ is 64.6% on average. The wrong detection of these estimation targets will lead to a decrease in usability. In contrast, in the case of Mixin, the F-measure of MR, LJ, and RJ are 86.0% on average, indicating that the wrong positives of these estimation targets are less than None. In the case of FT, the F-measure of MR, LJ, and RJ improved compared to None, but the average F-measure is 76.7%. Therefore, considering that it is difficult to distinguish between LJ and RJ, the estimation accuracy is 88.0% when they are considered as one estimation target. The estimation accuracy is 95.5% when MR, LJ, and RJ are considered as one estimation target. Therefore, we believe that a system with less misclassification in practical use can be realized using these three estimation targets as one label, such as jumping on one foot.

## 6. Experiments for Evaluating On-Device Performance

### 6.1. How to Verify the Performance on a Smartwatch

We implemented our method on a smartwatch to run on a device, and verified the effectiveness of our method in a real environment. We used Apple Watch as the smartwatch and Core ML for the model implementation. Core ML is a machine learning framework developed by Apple that is optimized for Apple’s hardware and can run prediction processing by machine learning models completely on-device. In Core ML, if we add a mlmodel file, the model format of Core ML, to Xcode, Xcode automatically generates the source code for use in the application. Applications can use the generated code to perform a prediction by using the model. Models from Python libraries, such as TensorFlow, can also be converted for Core ML. We can use Core ML Tools to convert the model built by Python libraries to the mlmodel format.

**Figure 8:**
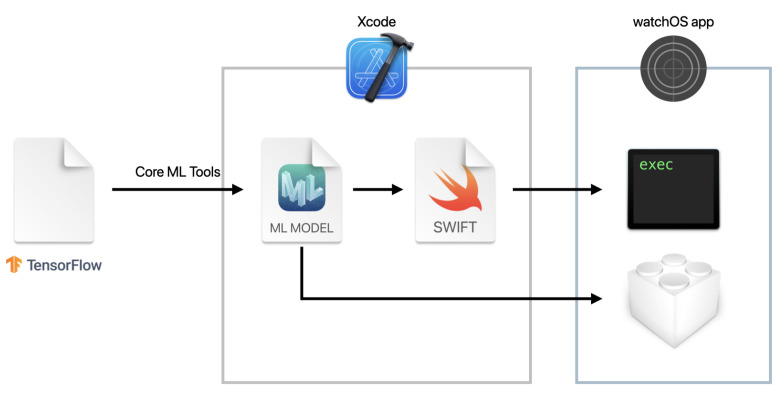
Conversion workflow for using a model on watchOS.

**Figure 9:**
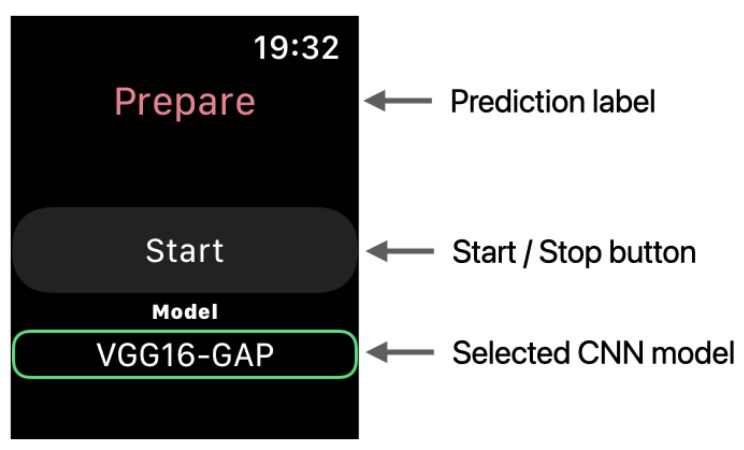
watchOS app used in the experiment.

We describe herein the watchOS App for evaluating the on-device performance of the models. Figure 8 illustrates the conversion workflow for using the created model on the Apple Watch. First, the model created by TensorFlow was converted to the mlmodel format using Core ML Tools. The mlmodel file was added to Xcode and used in the application. The application collected the acceleration data at 0.01 s intervals (100 Hz) using the Core Motion framework and performed inference on the model after collecting 64 samples in three axes. Figure 9 depicts a screenshot of the created watchOS App. The acceleration data were collected when the button was tapped. The model performed inference when the data satisfying the window size were collected.

We used the latency and the power consumption to evaluate the performance on the smartwatch. We also described the experiment for evaluating the on-device performance. Using the watchOS app that we created, we collected acceleration data and perform inference for 1 h. The app recorded the latency and the remaining battery power after each inference. We started the experiment with 100% battery. During the experiments, the Apple Watch was kept stationary with the app running. The model was evaluated based on the average inference time and the battery consumption when the acceleration data were collected, and the model inference was run for 1 h. The verification was performed on an Apple Watch SE with watchOS 7.3.2.

### 6.2. Results

Table 7 shows the mlmodel file size, average inference time, and battery consumption for each CNN model. The model size of our CNN model was the smallest compared to the other models because the filter size of the convolutional layer was small and the number of parameters was reduced by using GAP. In contrast, VGG16, which had the same structure for the feature extraction part, had the largest model size because the number of parameters was larger than our method due to the usage of fully-connected layers. In addition, Simple CNN showed the smallest inference time, which may be because it requires less processing for inference due to the small number of layers. However, in all models, the inference time was less than what it took to collect the acceleration data needed for inference (640 ms). Therefore, it is practical to run the model on the Apple Watch. In addition, the battery consumption for the acceleration data collection and inference was only approximately 7% at most after 1 h of use. Even if we assume that the amount of time spent on trampoline exercise per day is at most 1 h, it is still within the range of normal use, even with the limited battery capacity of the Apple Watch. However, note that these performance results were obtained using Apple Watch and Core ML. Core ML is built on top of Apple’s device-optimized low-level foundation of Accelerate, BNNS, and Metal Performance Shaders. Therefore, the performance obtained herein may not be achievable on other platforms; thus, verifying the performance on other platforms will be a future task.

## 7. Conclusions

In this study, we developed and evaluated a method for detecting the motions performed on a trampoline from the acceleration data obtained from a smartwatch. Accordingly, we proposed a method based on VGG16, an effective CNN model for activity recognition, and verified its effectiveness. Consequently, an accuracy that was 6% higher than the original VGG16 was achieved by using GAP. In addition, when we verified the ability of our method to classify actions in detail, we found it difficult to classify three types of jumps and marching. Marching was likely to misclassify one-legged jumps. We also evaluated the CNN models performance on an Apple Watch. Our method was found to have a small model size and was practical in terms of the inference time and the battery consumption. In the future, we would like to investigate the use of sensors not used in this study, as well as methods using dynamic window widths, and verify whether or not these methods can achieve a high classification accuracy. We would also like to evaluate the performance on platforms other than Apple Watch.

## Figures and Tables

**Table 1:** Model architecture.

Layer Type	Number of Filter	Shape of Output	Output Channels
Conv1D	16	192	16
Conv1D	16	192	16
MaxPooling1D	2	96	16
Conv1D	32	96	32
Conv1D	32	96	32
MaxPooling1D	2	48	32
Conv1D	64	48	64
Conv1D	64	48	64
Conv1D	64	48	64
MaxPooling1D	2	24	128
Conv1D	128	24	128
Conv1D	128	24	128
Conv1D	128	24	128
MaxPooling1D	2	12	128
Conv1D	128	12	128
Conv1D	128	12	128
Conv1D	128	12	128
MaxPooling1D	2	6	128
GAP	-	-	128
Softmax	-	-	6

**Table 2:** Subjects information.

ID	Age	Gender	Dominant	Height [cm]	Weight [kg]
A	23	Male	Right	163.0	52.0
B	22	Male	Right	169.5	55.0
C	23	Male	Right	161.0	54.0
D	24	Male	Right	173.0	55.0
E	22	Male	Right	165.0	75.0
F	23	Male	Right	170.5	60.0
G	22	Male	Right	170.0	76.5
H	23	Male	Right	170.0	55.0

**Table 3:** List of features.

Domain	Feature Name
Time	Mean
	Mean of absolute values
	Standard deviation
	Standard deviation of absolute values
	Minimum
	Maximum
	Root mean square
	1st quartile
	Median
	3rd quartile
	Interquartile range
	Correlation coefficient between axes
	Correlation coefficient of absolute values between axes
	Initial value in the frame
	Final value in the frame
	Intensity
	Skewness
	Kurtosis
	Zero-crossing rate
Frequency	Maximum
	Frequency of maximum
	2nd maximum
	Frequency of 2nd maximum
	Standard deviation
	1st quartile
	Median
	3rd quartile
	Interquartile range
	Correlation coefficient between axes

**Table 4:** Accuracy of each method for each subject.

Subject	RF	Simple CNN [14]	VGG16 [15]	Ours
A	74.4	73.3	76.2	**82.9**
B	77.3	**80.9**	71.1	80.7
C	57.6	66.7	59.3	**67.1**
D	**86.7**	72.4	68.0	76.0
E	75.6	82.0	75.6	**85.1**
F	58.0	63.6	63.3	**68.0**
G	63.3	82.2	82.4	**88.0**
H	82.4	76.7	**86.4**	82.4
Avg.	71.9	74.7	72.8	**78.8**

**Table 5:** Confusion Matrix of the RF.

Pre.\Cor.	ST	WK	MR	TJ	LJ	RJ	Precision [%]
ST	591	4	1	4	0	0	98.5
WL	7	551	39	3	0		91.7
MR	1	42	360	71	121	120	50.3
TJ	1	0	32	456	50	55	76.8
LJ	0	2	101	39	328	121	55.5
RJ	0	1	67	27	101	303	60.7
Recall [%]	98.5	91.8	60.0	76.0	54.7	50.5	71.9
F-measure [%]	98.5	91.8	54.8	76.4	55.1	55.1	

**Table 6:** Confusion matrix of our method.

Pre.\Cor.	ST	WL	MR	TJ	LJ	RJ	Precision [%]
ST	581	3	0	3	0	0	99.0
WL	8	578	65	3	2	5	87.4
MR	1	16	379	13	95	83	64.6
TJ	4	1	9	479	32	30	86.5
LJ	0	2	88	64	422	91	63.3
RJ	0	0	59	38	50	391	72.7
Recall [%]	97.8	96.3	63.2	79.8	70.3	65.2	78.8
F-measure [%]	98.4	91.7	63.9	83.0	66.6	68.7	

**Table 7:** Model size and performance on Apple Watch.

Model	Size [MB]	Time [ms/window]	Battery [%/h]
Simple CNN	4.0	8.1	7
VGG16	8.6	17.8	5
Ours	1.3	12.5	5

## Data Availability

The data used to support the findings of this study are available from the corresponding author upon request.
